# Temporal Trends in Janus Kinase Inhibitor (JAKi) Use Across Five European Countries: A DARWIN EU Network Study

**DOI:** 10.1002/pds.70502

**Published:** 2026-10-14

**Authors:** Xihang Chen, Andrei Barbulescu, George Corby, Saeed Hayati, Annika Jodicke, Toni Lehtonen, Hedvig Marie Egeland Nordeng, Alexandra Pacurariu, Gabriel Sanfélix‐Gimeno, Nhung T. H. Trinh, Katia Verhamme, Dina Vojinovic, Daniel Prieto‐Alhambra, Amy Shuk‐Man Lam, Edward Burn

**Affiliations:** ^1^ Pharmaco‐ and Device Epidemiology Group, Health Data Sciences, Nuffield Department of Orthopaedics, Rheumatology and Musculoskeletal Sciences (NDORMS) University of Oxford Oxford UK; ^2^ Data Analytics and Methods Task Force Real World Evidence Department European Medicines Agency Amsterdam the Netherlands; ^3^ Pharmacoepidemiology and Drug Safety Research Group, Department of Pharmacy University of Oslo Oslo Norway; ^4^ Unit of Data Production and Methods. Data and Analytics Finnish Institute for Health and Welfare Helsinki Finland; ^5^ Fundació Per al Foment de la Investigació Sanitària i Biomèdica de la Comunitat Valenciana Valencia Spain; ^6^ Erasmus Medical Center Rotterdam CA the Netherlands; ^7^ IQVIA Amsterdam CT the Netherlands

**Keywords:** drug utilisation, JAK inhibitor, JAKi switching, JAKi treatment duration, Janus kinase inhibitor

## Abstract

**Introduction:**

Janus kinase inhibitors (JAKi) are increasingly used for autoimmune conditions despite existing safety concerns. This study aims to estimate the incidence of new JAKi use over time and characterise users in Europe to support future safety studies.

**Methods:**

The study included individuals from five European data sources within the DARWIN EU network: FinOMOP‐HILMO (Finland; primary and secondary care), IPCI (the Netherlands; primary), IQVIA DA Germany (Germany; primary), NLHR (Norway; primary and secondary), VID (Spain; primary and secondary) from 1 January 2017. Annual incidence of JAKi (abrocitinib, baricitinib, filgotinib, tofacitinib, upadacitinib) was up to 31 December 2023. Patient characteristics and treatment duration were described.

**Results:**

Incidence of JAKi use increased over time, with transient declines during the COVID‐19 pandemic. Initiators, except for abrocitinib, were predominantly aged 41–60 years. Rheumatoid arthritis was the most common indicated condition, except for abrocitinib, for which atopic dermatitis predominated. Median treatment duration was generally less than 1 year. Tofacitinib and baricitinib had the lowest proportions of prior JAKi use, whereas up to 17% and 16% of upadacitinib and filgotinib initiators had received prior treatment with another JAKi. Findings were consistent across data sources, although incidence rates varied according to the healthcare sector captured.

**Conclusions:**

Incidence of JAKi use generally increased, with tofacitinib being the most initiated JAKi. Treatment durations were relatively short and switching between JAKi was common. The current study provides an overview of indication distribution and characterises JAKi treatment, while offering a framework for future research.

## Key Points

- JAKi initiation increased across five European data sources from 2017 to 2023, with temporary reductions in some settings during the pandemic.
- Tofacitinib was the most frequently initiated JAKi, whereas abrocitinib had the lowest observed use.
- Most initiators were aged 41–60 years, and rheumatoid arthritis was the most commonly recorded indicated condition, except for abrocitinib.
- Recorded treatment episodes were generally shorter than 1 year where duration could be estimated.
- Prior use of another JAKi was observed, supporting the need to account for within‐class switching in future studies.

## Plain Language Summary

Janus kinase inhibitors, also known as JAK inhibitors or JAKi, are medicines used to treat autoimmune and inflammatory conditions, but concerns remain about their safety. This study looked at how often people started JAKi treatment and what kinds of patients used these medicines across five European healthcare data sources in Finland, the Netherlands, Germany, Norway and Spain. The study included five JAKi: abrocitinib, baricitinib, filgotinib, tofacitinib and upadacitinib and covered the period from 2017 to 2023. Overall, new JAKi use increased over time, although some temporary decreases were seen during the COVID‐19 pandemic. Tofacitinib was the most commonly started JAKi, while abrocitinib was used least often. Most people starting JAKi, except those starting abrocitinib, were aged 41–60 years and rheumatoid arthritis was the most common recorded condition linked to treatment. For abrocitinib, atopic dermatitis was the most common condition. Where treatment duration could be measured, treatment episodes were usually shorter than 1 year. Some patients had previously used another JAKi, showing that switching between medicines in this class occurs in routine care. These findings describe how JAKi are used in Europe and can help plan future safety studies.

## 1 Introduction

Janus kinase inhibitors (JAKi) have been gaining popularity for treating several autoimmune conditions [1]. The first approved JAKi for a rheumatological condition was tofacitinib for moderately or severely active rheumatoid arthritis uncontrolled by methotrexate in adults [2]. The Oral Rheumatoid Arthritis Trial (ORAL) Surveillance found a higher risk of major adverse cardiovascular events (MACE), cancer and adjudicated opportunistic infection with tofacitinib compared to tumour necrosis factor (TNF) inhibitors in patients aged ≥ 50 years with at least one cardiovascular risk factor [3]. Evidence on the safety of JAKi in other indications, including psoriatic arthritis, ulcerative colitis and atopic dermatitis, remains limited by short follow‐up and small sample sizes [4, 5, 6, 7], especially that these new indications have only been recently approved (Table S1). Therefore, a study conducted within a federated network such as the Data Analysis and Real World Interrogation Network (DARWIN EU) may offer a viable solution. DARWIN EU is an initiative of the European Medicines Agency (EMA) that enables the analysis of harmonised real‐world healthcare data across multiple countries through a federated infrastructure. The current study aims to identify and characterise eligible JAKi users within the DARWIN EU network, thereby informing the feasibility of future safety studies.

## 2 Methods

### 2.1 Study Design, Setting and Data Sources

This was a retrospective, observational population‐based cohort study with two aims: to estimate the incidence of JAKi uptake in the general population and to describe the characteristics of JAKi initiators. The study used five data sources within the DARWIN EU network [8], all mapped to the Observational Medical Outcomes Partnership (OMOP) Common Data Model (CDM) [8]: Finnish Care Register for Health Care (FinOMOP‐HILMO), Finland; Integrated Primary Care Information (IPCI), the Netherlands; IQVIA Disease Analyzer Germany (IQVIA DA Germany), Germany; Norwegian Linked Health Registry (NLHR), Norway and Valencia Health System Integrated Dataset (VID), Spain. Details of the electronic health record (EHR) data sources are provided in Table S2.

### 2.2 Study Period, Study Participants and Follow‐Up

#### 2.2.1 Study Period

The study period ran from 1 January 2017 to the latest data availability for each data source, although follow‐up definitions differed across databases. In IQVIA DA Germany, follow‐up was defined from the earliest to latest recorded event; therefore, individuals without encounters near the data cut may not be captured as active, potentially causing an artificial decline in the denominator population while incident user counts remain stable. To reduce this bias, IQVIA DA Germany follow‐up was censored 6 months before the data cut, in December 2023. In other data sources, follow‐up was defined from registration to deregistration (FinOMOP‐HILMO, IPCI, VID) or from birth to end of data collection (NLHR) (Table S1). Latest data availability was October 2024 for FinOMOP‐HILMO, June 2024 for IPCI, December 2023 for NLHR and December 2021 for VID.

#### 2.2.2 Incidence of JAKi Uptake

For this objective, individuals were eligible if they were present in the database during the study period and had ≥ 365 days of prior observation. Annual incidence was estimated for overall JAKi use and for each of the five active drugs: abrocitinib, baricitinib, filgotinib, tofacitinib and upadacitinib. Overall JAKi use was defined as the first use of any JAKi, while the incidence of each active drug was estimated by considering only the use of that particular active drug. Individuals were required to have full‐year observation to ensure consistent observation time across data sources for annual incidence estimation. For incidence of first‐ever JAKi use, individuals with any prior JAKi exposure prior to the study period were excluded. For drug‐specific analyses, individuals with prior exposure to the same drug before study start date were excluded.

In NLHR, as drug records were only available from 2018, thus incidence analysis started from 2019 to avoid misinterpreting prevalent uses as incident ones.

#### 2.2.3 Characterisation of JAKi Initiators

JAKi initiators were characterised at the drug level. Eligible participants had ≥ 365 days of prior observation and no prior exposure to the same drug and initiated treatment during the study period. Characterisation was given by baseline demographics, presence of indicated conditions for JAKi, duration of JAKi treatment and prior exposure to other JAKi.

### 2.3 Exposure, Outcome and Covariate

For incidence estimation of JAKi uptake, analysis included all eligible individuals in the data sources without exposure requirement. Outcome was defined as first use of JAKi, overall and for each of the five drugs (abrocitinib, baricitinib, filgotinib, tofacitinib and upadacitinib). Outcome of overall JAKi use was defined as the first use of any of the five included JAKi ingredients.

For characterisation of JAKi initiators, analysis included individuals with exposure to each of the five JAKi. Covariates used for characterisation included age and sex at the index date, indicated conditions (alopecia areata, atopic dermatitis, axial spondylitis, inflammatory bowel disease, juvenile arthritis, juvenile idiopathic arthritis, psoriatic arthritis and rheumatoid arthritis) and JAKi treatment duration. As treatment indication is not explicitly recorded in the data sources, indicated conditions were inferred based on the presence of relevant diagnoses recorded prior to or on the index date. Analyses were conducted as overall and stratified by indicated conditions. The process of identifying JAKi and indicated conditions followed the DARWIN EU phenotyping procedure [9], with code lists available in Table S3.

### 2.4 Statistical Methods

#### 2.4.1 Incidence of JAKi Uptake

The annual incidence of JAKi use was calculated as the number of individuals with a first prescription or dispensation of a JAKi per 100 000 person‐years (pys) among the at‐risk population during the follow‐up period. Incidence rates were reported with 95% exact Poisson confidence intervals (CIs). All analyses were performed using the R package *IncidencePrevalence* v1.2.0 [10]. Additional analysis on drug‐specific incidence was conducted in individuals without prior exposure to any JAKi.

#### 2.4.2 Characterisation of JAKi Initiators

Characterisation of JAKi users were given by each of the five individual JAKi drug. Demographics (age and sex) and presence of indicated conditions were given at baseline. Treatment duration was derived from the OMOP CDM drug exposure table using recorded start and end dates; prescription or dispensation records were concatenated into a single drug era if gaps between exposures were ≤ 90 days, starting from the first prescription within the study period. Duration of JAKi use was defined as the length of the first drug era during the study period, expressed in months. Characteristics of JAKi users were presented as count (percentage) or median (interquartile range, IQR) as appropriate. Analyses were performed using the R packages CohortCharacteristics v0.4.0 [11] and DrugUtilisation v0.8.2 [12].

Analyses with counts less than five were reported as ‘< 5’ for privacy reasons.

## 3 Results

All results presented in the Results section can be accessed via the interactive R Shiny dashboard: EUPAS1000000424.

After applying exclusion criteria (Table S4), a total of 6 133 929 individuals from FinOMOP‐HILMO, 2 016 752 individuals from IPCI, 14 311 724 individuals from IQVIA DA Germany, 5 834 880 individuals from NLHR and 1 573 602 individuals from VID were eligible in the denominator population for the incidence analyses.

### 3.1 Incidence of JAKi Uptake

A total number of 4216 JAKi users from FinOMOP‐HILMO, 394 JAKi users from IPCI, 3893 JAKi users from IQVIA DA Germany, 3625 JAKi users from NLHR and 273 JAKi users from VID were identified for the incidence of overall JAKi use analysis (Table 1). Considering each of the five included JAKi drugs, tofacitinib had the highest number of initiators while abrocitinib had the lowest (Table S5).

**TABLE 1:** Number of JAKi initiators identified for incidence analysis.

Year^a^	Data sources				
	FinOMOP‐HILMO	IPCI	IQVIA‐DA Germany	NLHR	VID
2017	172	22	178	—	—
2018	773	43	385	—	—
2019	702	52	555	564	70
2020	490	57	742	394	70
2021	656	67	904	638	133
2022	649	86	1129	962	—
2023	774	67	—	1067	—
Overall	4216	394	3893	3625	273

Abbreviations: FinOMOP‐HILMO = Finnish Care Register for Health Care; IPCI = Integrated Primary Care Information; IQVIA DA Germany = IQVIA Disease Analyzer Germany; JAKi = Janus kinase inhibitor; NLHR = Norwegian Linked Health Registry; VID = Valencia Health System Integrated Dataset.

^a^ Number of JAKi initiators identified for incidence analysis considered full‐year contribution across all data sources and drug record availability since 2018 in NLHR.

The incidence of overall JAKi use increased from 2017 to 2023, with a transient reduction in 2020 in FinOMOP‐HILMO and NLHR (Figure 1). Incidence rates rose in FinOMOP‐HILMO from 3.1 to 14.0/100 000 pys, in IQVIA DA Germany from 2.3 to 15.6 and in VID from 4.7 to 8.9. In NLHR, incidence dropped from 10.7 in 2019 to 7.4 in 2020, before rising to 21.2 in 2023.

**FIGURE 1:**
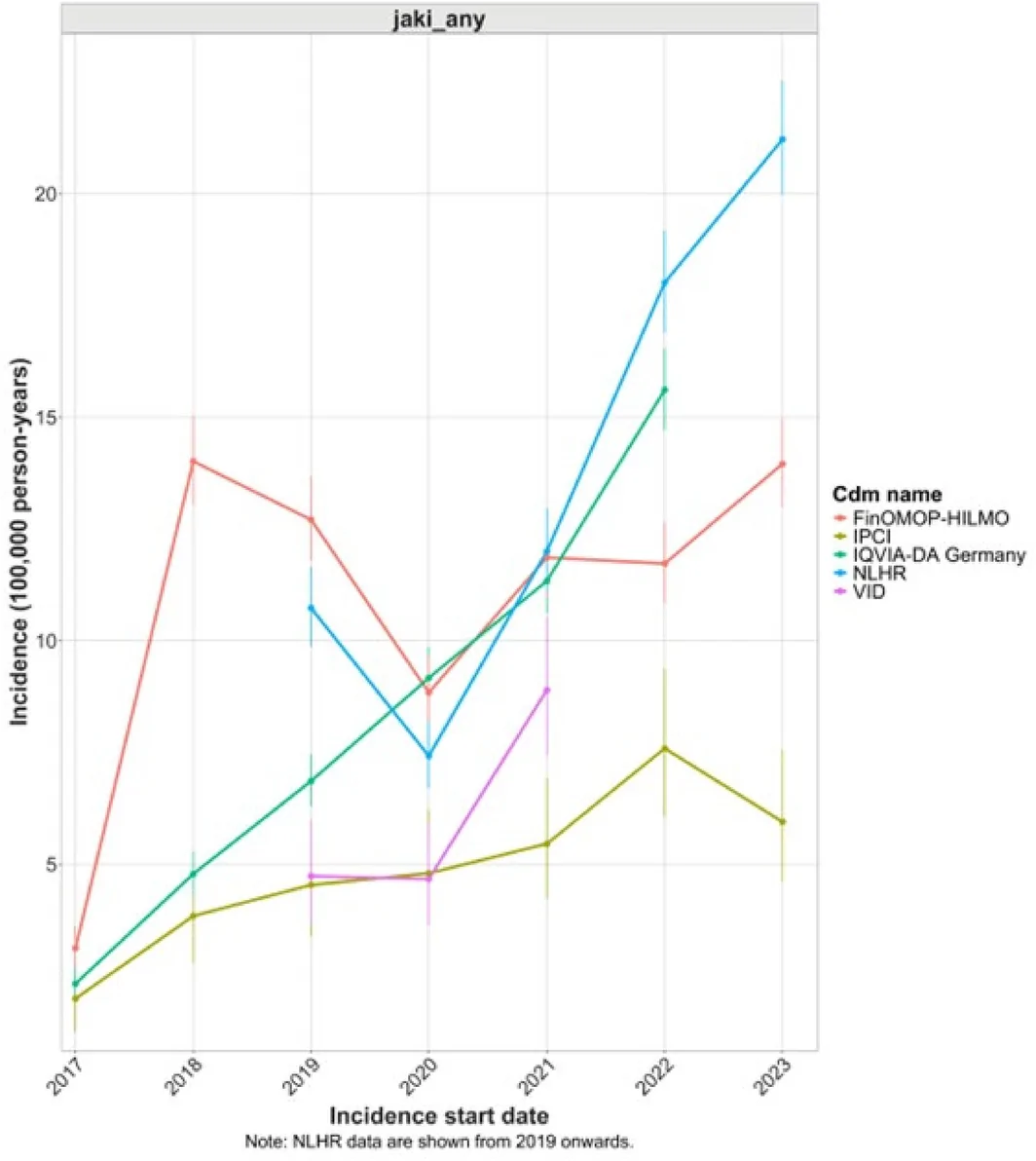
Incidence of any JAKi use among the individuals without prior exposure to any JAKi. FinOMOP‐HILMO = Finnish Care Register for Health Care; IPCI = Integrated Primary Care Information; IQVIA DA Germany = IQVIA Disease Analyzer Germany; NLHR = Norwegian Linked Health Registry; VID = Valencia Health System Integrated Dataset.

Incidence was assessed for each individual JAKi among individuals without prior exposure to the same JAKi (Figure 2). Early increasing trends were seen for baricitinib and tofacitinib, while filgotinib and upadacitinib showed growth from 2020 onwards. When considering drug‐specific incidence among individuals with no prior use of any JAKi in the additional analysis (Figure S1), the observed trends mirrored those seen in individuals with no prior exposure to respective individual JAKi.

**FIGURE 2:**
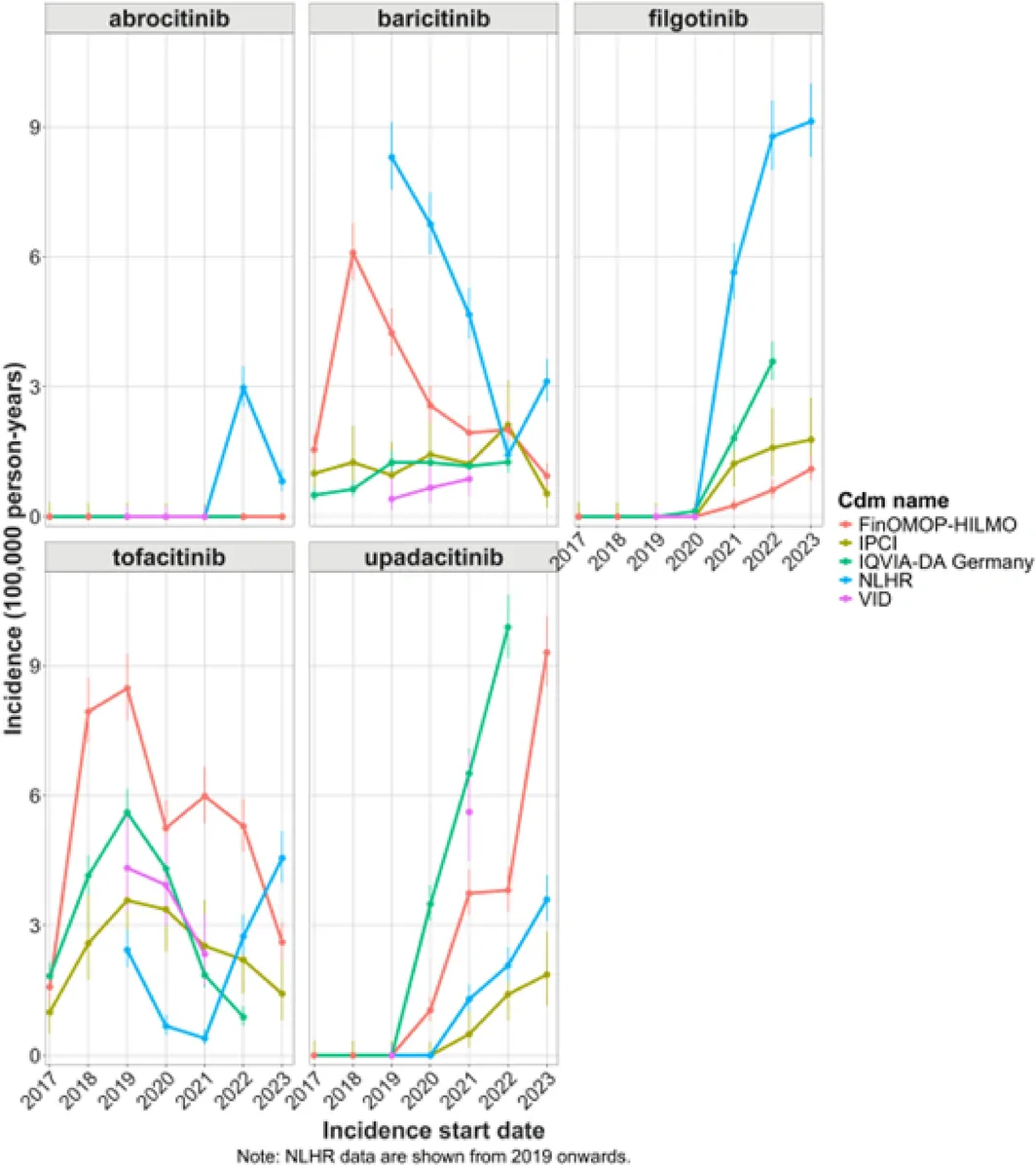
Incidence of each JAKi ingredient use among the individuals without prior exposure to the same JAKi ingredient. FinOMOP‐HILMO = Finnish Care Register for Health Care; IPCI = Integrated Primary Care Information; IQVIA DA Germany = IQVIA Disease Analyzer Germany; NLHR = Norwegian Linked Health Registry; VID = Valencia Health System Integrated Dataset.

### 3.2 Characterisation of JAKi Initiators

Median age at initiation was lowest for abrocitinib, with most users starting before age 40; initiators of other JAKi were mostly aged 41–60 years (Table 2). Where counts were sufficient, median age at initiation was higher in IQVIA DA Germany than in other data sources. In data sources with sufficient treatment‐duration data, median durations were generally similar across JAKi, ranging from 3 to 9 months for filgotinib to 4–10 months for abrocitinib. However, durations varied within data sources: IQRs ranged from 3 to 14 months for baricitinib to 3–21 months for tofacitinib in IQVIA DA Germany and from 2 to 11 months for upadacitinib to 4–29 months for baricitinib in NLHR.

**TABLE 2:** Characteristics of JAKi initiators at the individual drug level.

JAKi drug	Variable name	Variable level	Data sources				
			FinOMOP‐HILMO	IPCI	IQVIA DA Germany	NLHR	VID
Abrocitinib	Number of initiators		—	16	—	298	—
	Sex	Female (%)	—	7 (43.8%)	—	121 (40.6%)	—
	Age	Median [Q25–Q75]	—	34 [25–46]	—	32 [25–46]	—
	Treatment duration	Median [Q25–Q75] (months)	—	4 [1–8]	—	10 [4–14]	—
	Indications	Alopecia areata (%)	—	0 (0.0%)	—	14 (4.7%)	—
		Atopic dermatitis (%)	—	5 (31.3%)	—	297 (99.7%)	—
		Axial spondylitis (%)	—	0 (0.0%)	—	0 (0.0%)	—
		Inflammatory bowel disease (%)	—	< 5	—	< 5	—
		Juvenile arthritis (%)	—	0 (0.0%)	—	< 5	—
		Juvenile idiopathic arthritis (%)	—	0 (0.0%)	—	0 (0.0%)	—
		Psoriatic arthritis (%)	—	< 5	—	< 5	—
		Rheumatoid arthritis (%)	—	0 (0.0%)	—	15 (5.0%)	—
	Prior exposure of other JAKi	Baricitinib	—	< 5	—	97 (32.6%)	—
		Filgotinib	—	0 (0.0%)	—	0 (0.0%)	—
		Tofacitinib	—	0 (0.0%)	—	0 (0.0%)	—
		Upadacitinib	—	< 5	—	< 5	—
Baricitinib	Number of initiators		1205	110	630	1538	30
	Sex	Female (%)	957 (79.4%)	80 (72.7%)	509 (80.8%)	1048 (68.1%)	30 (100.0%)
	Age	Median [Q25–Q75]	57 [44–67]	58 [49–69]	71 [61–78]	54 [38–66]	50 [45–54]
	Treatment duration	Median [Q25–Q75] (Months)	^a^	4 [1–18]	6 [3–14]	9 [4–29]	^a^
	Indications	Alopecia areata (%)	34 (2.8%)	< 5	12 (1.9%)	63 (4.1%)	< 5
		Atopic dermatitis (%)	132 (11.0%)	10 (9.1%)	36 (5.7%)	560 (36.4%)	< 5
		Axial spondylitis (%)	16 (1.3%)	22 (20.0%)	5 (0.8%)	43 (2.8%)	0 (0.0%)
		Inflammatory bowel disease (%)	26 (2.2%)	< 5	6 (1.0%)	35 (2.3%)	0 (0.0%)
		Juvenile arthritis (%)	99 (8.2%)	0 (0.0%)	< 5	128 (8.3%)	< 5
		Juvenile idiopathic arthritis (%)	62 (5.2%)	0 (0.0%)	< 5	34 (2.2%)	0 (0.0%)
		Psoriatic arthritis (%)	34 (2.8%)	< 5	16 (2.5%)	129 (8.4%)	0 (0.0%)
		Rheumatoid arthritis (%)	1057 (87.7%)	50 (45.5%)	539 (85.6%)	1109 (72.1%)	28 (93.3%)
	Prior exposure of other JAKi	Abrocitinib	0 (0.0%)	0 (0.0%)	0 (0.0%)	37 (2.4%)	0 (0.0%)
		Filgotinib	—	0 (0.0%)	6 (1.0%)	18 (1.2%)	0 (0.0%)
		Tofacitinib	118 (9.8%)	8 (7.3%)	39 (6.2%)	179 (11.6%)	0 (0.0%)
		Upadacitinib	13 (1.1%)	—	18 (2.9%)	5 (0.3%)	0 (0.0%)
Filgotinib	Number of initiators		156	72	752	1560	< 5
	Sex	Female (%)	100 (64.1%)	45 (62.5%)	568 (75.5%)	1058 (67.8%)	< 5
	Age	Median [Q25–Q75]	47 [37–60]	57 [48–62]	60 [51–68]	51 [36–64]	< 5
	Treatment duration	Median [Q25—Q75] (months)	^a^	3 [2–11]	9 [4–19]	8 [3–16]	^a^
	Indications	Alopecia areata (%)	0 (0.0%)	0 (0.0%)	< 5	51 (3.3%)	0 (0.0%)
		Atopic dermatitis (%)	9 (5.8%)	< 5	9 (1.2%)	278 (17.8%)	0 (0.0%)
		Axial spondylitis (%)	0 (0.0%)	12 (16.7%)	15 (2.0%)	71 (4.6%)	0 (0.0%)
		Inflammatory bowel disease (%)	74 (47.4%)	14 (19.4%)	114 (15.2%)	499 (32.0%)	0 (0.0%)
		Juvenile arthritis (%)	14 (9.0%)	0 (0.0%)	7 (0.9%)	133 (8.5%)	0 (0.0%)
		Juvenile idiopathic arthritis (%)	9 (5.8%)	0 (0.0%)	< 5	35 (2.2%)	0 (0.0%)
		Psoriatic arthritis (%)	5 (3.2%)	0 (0.0%)	35 (4.7%)	168 (10.8%)	0 (0.0%)
		Rheumatoid arthritis (%)	80 (51.3%)	23 (31.9%)	616 (81.9%)	1150 (73.7%)	0 (0.0%)
	Prior exposure of other JAKi	Abrocitinib	0 (0.0%)	0 (0.0%)	0 (0.0%)	0 (0.0%)	0 (0.0%)
		Baricitinib	25 (16.0%)	5 (6.9%)	25 (3.3%)	184 (11.8%)	0 (0.0%)
		Tofacitinib	25 (16.0%)	7 (9.7%)	100 (13.3%)	191 (12.2%)	0 (0.0%)
		Upadacitinib	17 (10.9%)	0 (0.0%)	102 (13.6%)	16 (1.0%)	0 (0.0%)
Tofacitinib	Number of initiators		2129	210	1583	1472	160
	Sex	Female (%)	1322 (62.1%)	136 (64.8%)	1154 (72.9%)	1028 (69.8%)	160 (100.0%)
	Age	Median [Q25–Q75]	50 [34–61]	56 [45–66]	58 [50–67]	54 [40–64]	48 [41–53]
	Treatment duration	Median [Q25–Q75] (months)	^a^	4 [1–11]	7 [3–21]	7 [3–18]	^a^
	Indications	Alopecia areata (%)	6 (0.3%)	< 5	27 (1.7%)	44 (3.0%)	12 (7.5%)
		Atopic dermatitis (%)	119 (5.6%)	10 (4.8%)	10 (0.6%)	207 (14.1%)	< 5
		Axial spondylitis (%)	67 (3.2%)	64 (30.4%)	53 (3.4%)	153 (10.4%)	8 (5.0%)
		Inflammatory bowel disease (%)	681 (32.0%)	29 (13.8%)	231 (14.6%)	235 (16.0%)	13 (8.1%)
		Juvenile arthritis (%)	242 (11.3%)	0 (0.0%)	19 (1.2%)	157 (10.7%)	< 5
		Juvenile idiopathic arthritis (%)	138 (6.4%)	0 (0.0%)	< 5	36 (2.4%)	0 (0.0%)
		Psoriatic arthritis (%)	339 (15.9%)	< 5	268 (16.9%)	287 (19.5%)	42 (26.3%)
		Rheumatoid arthritis (%)	1202 (56.5%)	90 (42.9%)	1153 (72.8%)	1290 (87.6%)	103 (64.3%)
	Prior exposure of other JAKi	Abrocitinib	0 (0.0%)	0 (0.0%)	0 (0.0%)	0 (0.0%)	0 (0.0%)
		Baricitinib	54 (2.5%)	7 (3.3%)	19 (1.2%)	48 (3.3%)	0 (0.0%)
		Filgotinib	< 5	< 5	< 5	32 (2.2%)	0 (0.0%)
		Upadacitinib	20 (0.9%)	0 (0.0%)	17 (1.1%)	12 (0.8%)	< 5
Upadacitinib	Number of initiators		1296	86	2534	544	115
	Sex	Female (%)	801 (61.8%)	54 (62.8%)	1683 (66.4%)	344 (63.2%)	115 (100.0%)
	Age	Median [Q25–Q75]	47 [35–58]	48 [34–59]	57 [47–64]	49 [36–60]	49 [44–54]
	Treatment duration	Median [Q25—Q75] (months)	^a^	5 [2–11]	9 [3–20]	5 [2–11]	^a^
	Indications	Alopecia areata (%)	9 (0.7%)	0 (0.0%)	42 (1.7%)	20 (3.7%)	6 (5.2%)
		Atopic dermatitis (%)	197 (15.2%)	25 (29.1%)	210 (8.3%)	159 (29.2%)	7 (6.1%)
		Axial spondylitis (%)	143 (11.0%)	15 (17.4%)	197 (7.8%)	93 (17.1%)	5 (4.4%)
		Inflammatory bowel disease (%)	373 (28.8%)	14 (16.3%)	281 (11.1%)	183 (33.6%)	5 (4.4%)
		Juvenile arthritis (%)	81 (6.3%)	0 (0.0%)	20 (0.8%)	42 (7.7%)	< 5
		Juvenile idiopathic arthritis (%)	53 (4.1%)	0 (0.0%)	< 5	9 (1.7%)	< 5
		Psoriatic arthritis (%)	174 (13.4%)	0 (0.0%)	473 (18.7%)	241 (44.3%)	10 (8.7%)
		Rheumatoid arthritis (%)	708 (54.6%)	19 (22.1%)	1556 (61.4%)	380 (69.9%)	95 (82.6%)
	Prior exposure of other JAKi	Abrocitinib	0 (0.00%)	< 5	0 (0.00%)	21 (3.9%)	0 (0.00%)
		Baricitinib	140 (10.8%)	9 (10.4%)	55 (2.2%)	70 (12.9%)	< 5
		Filgotinib	8 (0.6%)	5 (5.8%)	51 (2.0%)	93 (17.1%)	0 (0.0%)
		Tofacitinib	186 (14.4%)	13 (15.1%)	286 (11.3%)	78 (14.3%)	20 (17.4%)

Abbreviations: FinOMOP‐HILMO = Finnish Care Register for Health Care; IPCI = Integrated Primary Care Information; IQVIA DA Germany = IQVIA Disease Analyzer Germany; JAKi = Janus kinase inhibitor; NLHR = Norwegian Linked Health Registry; VID = Valencia Health System Integrated Dataset.

^a^ Treatment duration could not be estimated in FinOMOP‐HILMO and VID due to limited data quality on drug records.

The majority of JAKi initiators had a history of rheumatoid arthritis as their likely indication, except for abrocitinib where 31.3% (IPCI) and 99.7% (NLHR) of abrocitinib initiators had a record of atopic dermatitis, with other commonly recorded indications for JAKi including inflammatory bowel disease, psoriatic arthritis and axial spondylitis.

The proportion of initiators with prior exposure to another JAKi varied by drug. Tofacitinib and baricitinib had the lowest proportions, whereas up to 17% of upadacitinib and 16% of filgotinib initiators had previously received another JAKi. Among initiators with rheumatoid arthritis, prior JAKi use ranged from 1% to 30% for filgotinib and 3%–37% for upadacitinib.

## 4 Discussion

Using five European data sources, this study estimated the incidence of JAKi use and characterised initiators across countries. Tofacitinib was the most frequently initiated JAKi, while abrocitinib had the lowest uptake. Incidence of new JAKi use increased steadily since 2017, with uptake patterns aligning with EMA approval dates (Table S1), alongside a transient decline during the COVID‐19 pandemic. Initiators of baricitinib, filgotinib, tofacitinib and upadacitinib were predominantly middle‐aged and had rheumatoid arthritis, whereas abrocitinib initiators were younger and had atopic dermatitis. Treatment duration was generally short and within‐class switching was common. This study outlines indication distribution and characterises JAKi use, providing a foundation for future research.

JAKi has become an important treatment option for autoimmune diseases in patients with an incomplete response to initial therapy. A 2024 review by Szekanecz et al. [13] summarised the vast evidence on efficacy and safety, highlighted outstanding clinical questions and underscored the role of JAKi in patients with active uncontrolled inflammation, supported by favourable number‐needed‐to‐treat and benefit–risk ratios in comparative trials. Loss of response over time has been the main reason for switching from TNF inhibitors to JAKi [14], while approximately half of JAKi use has been attributed to the convenience of oral administration compared with other advanced therapies (e.g., TNF inhibitors and non‐TNF biologics). These findings suggested the therapeutic advantage and role of JAKi in the treatment of autoimmune diseases.

However, safety concerns, including increased risks of MACE, serious infections and malignancy, were identified in the ORAL Surveillance trial of tofacitinib in patients with rheumatoid arthritis [3]. Following a review of the available evidence [15], the EMA recommended that JAKi should be used only if no suitable treatment alternatives are available in patients aged ≥ 65 years, current or past long‐term smokers and those at increased risk of cardiovascular events or malignancy [16]. Although the evidence was primarily derived from studies of tofacitinib and baricitinib in rheumatoid arthritis populations, these recommendations were applied across all JAKi and approved indications. Generating evidence for newer JAKi across broader indicated populations is therefore important. Multinational network studies using multiple data sources can increase sample size and event numbers, improve statistical power and enable more comprehensive safety assessment. By characterising JAKi use across countries and data sources, this study informs the feasibility and design of future safety studies within the DARWIN EU network. JAKi have been increasingly used across European countries despite the limited literature on their utilisation. A drug utilisation study from Italy reported that baricitinib was the most commonly used JAKi among patients with rheumatoid arthritis during 2018–2019 [17]. The same study also showed that JAKi were predominantly used in later lines of treatment, with 31.1% of users initiating as second‐line, 33.1% as third‐line and 27.8% as fourth‐line therapy. A national Polish database study reported increasing JAKi adoption in autoimmune disease management following approval for various indications, with growing market share, particularly, in rheumatoid arthritis [18]. This growth underscores the importance of evaluating and understanding the use of JAKi across various settings and localities, including its potential economic impact on healthcare expenditures. Moreover, JAKi has also been commonly prescribed as third‐line treatment for patients with severe alopecia areata [19], suggesting that their use had generally been reserved for cases with uncontrolled disease. To our knowledge, no drug utilisation study has been conducted on JAKi use across multiple databases from different European countries, highlighting the importance of this study in understanding JAKi utilisation in Europe. Patient characteristics aligned with the indications of different JAKi. Rheumatoid arthritis was the most commonly recorded indication among initiators of baricitinib, filgotinib, tofacitinib and upadacitinib, who were mostly female and aged 41–60 years, consistent with the general demographics of rheumatoid arthritis [20]. Conversely, abrocitinib is indicated for atopic dermatitis. As atopic dermatitis affects males and females more evenly and typically presents at a younger age, abrocitinib initiators showed a more balanced sex distribution and were generally younger, consistent with the epidemiology of the disease [21]. JAKi use was observed across multiple approved indications, supporting the feasibility of utilisation studies beyond rheumatoid arthritis and informing future safety research. Age patterns across indication‐specific cohorts were consistent with the epidemiology of the indicated conditions, serving as a data quality and consistency check. This study also included JAKi users across a wide age range, including older adults, middle‐aged individuals and younger users aged ≤ 40 years.

In contrast, the ORAL Surveillance study [3], that focused on patients with rheumatoid arthritis, aged 50 or above. Therefore, this study provides a broader and more comprehensive examination of the JAKi usage in a more diverse population.

Overall, treatment duration of JAKi was short, with median durations consistently less than 1 year across the two databases where these analyses were feasible. This finding aligns with a meta‐analysis of tofacitinib effectiveness in patients with ulcerative colitis, which included 19 observational studies (median follow‐up: 7–44 weeks) and reported treatment discontinuation rates ranging from 12% to 56% [22]. The treatment duration in the present study was comparatively shorter than that from the ORAL Surveillance post‐authorisation safety study, of which the median duration of tofacitinib use in rheumatoid arthritis patients was 44 months [3]. Lack of efficacy and loss of treatment response have been widely reported as the main reasons for the JAKi discontinuation [23, 24]. Future JAKi safety studies should take this into account during study design. The relatively short treatment duration observed also warrants cautious interpretation of safety findings, particularly in relation to time at risk. Importantly, adverse events associated with JAKi should be evaluated in light of their use in relation to the pathophysiology and expected timing of outcome development within the actual treatment duration. For example, post hoc analyses of the ORAL Surveillance study demonstrated that the risk of non‐serious infections and herpes zoster was highest within the first 6 months of tofacitinib use [25] whereas the risk of malignancy increased after 18 months of JAKi treatment [26]. Nevertheless, the relatively short JAKi treatment durations observed in the present study should not be overinterpreted. The available data did not allow us to determine whether the end of an observed treatment episode represented true treatment discontinuation or whether individuals subsequently switched to another JAKi. Furthermore, information on the reasons for treatment discontinuation was not available. These factors should therefore be considered when interpreting the observed treatment durations and when designing future studies using these data sources.

We also observed JAKi switching in this study. Among the JAKi initiators with rheumatoid arthritis, up to 30% of filgotinib initiators and up to 37% of upadacitinib initiators received prior treatment with other JAKi. In real‐world practice, switching between JAKi has been reported across multiple disease populations, including rheumatoid arthritis [23, 27], ulcerative colitis [28], alopecia areata [29] and atopic dermatitis [30]. Evidence suggests that switching (or ‘JAKi cycling’) may be a viable strategy. Within the international JAK‐pot collaboration of 17 national registries, patients with rheumatoid arthritis who switched to another JAKi after treatment failure had similar crude treatment retention rates compared with those switching to a biologic. However, when considered also covariates, patients switching to another JAKi were associated with lower drug discontinuation rates compared with those switching to a biologic (hazard ratio for drug discontinuation 0.82, 95% CI: 0.68–0.99) [31]. A Swiss observational study [32] also showed a lower drug discontinuation rate among patients switching to another JAKi than among those switching to TNF inhibitor (hazard ratio for drug discontinuation 0.48, 95% CI: 0.30–0.76). These findings highlight the clinical relevance of JAKi switching and underscore the need to account for such treatment patterns when designing future JAKi safety studies. Prior JAKi exposure may be associated with treatment history, disease severity, treatment response or other patient characteristics that could also be related to subsequent safety outcomes and may, therefore, introduce confounding when comparing individual JAKi or treatment strategies. Future comparative safety studies should carefully characterise prior JAKi exposure and treatment sequencing and consider these factors in study design and analytical approaches. Further investigation of longitudinal treatment sequences would also help to better characterise JAKi cycling and its role in real‐world clinical practice.

This study has limitations. First, it used routinely collected healthcare data and is therefore subject to data quality issues. Exposure misclassification is possible because prescription or dispensation records do not necessarily indicate that medication was taken. However, established methods were used to estimate treatment duration by concatenating consecutive prescriptions and accounting for refill gaps [33]. Treatment duration was not estimated in VID or FinOMOP‐HILMO because reliable prescription end dates were unavailable. Interpretation of treatment duration and switching should also consider differences in JAKi approval dates, as newer drugs had shorter potential follow‐up and observation time. More generally, heterogeneous follow‐up periods across the databases may limit the comparability of results between data sources, particularly for analyses requiring longitudinal observation. Prescribing indication was not explicitly recorded, so it was inferred from eight pre‐specified diagnoses recorded on or before treatment initiation; potential indications may therefore be incompletely captured. Misclassification of treatment indication was possible as the identified diagnosis might not be directly linked to individual prescription or dispensation records. Consequently, although a recorded diagnosis may represent a potential indication for JAKi treatment, it cannot be confirmed as the true indication for a specific JAKi initiation. Data quality also varied across sources. A diagnosis compatible with a JAKi indication was identified in approximately 90% of new users in all databases except IPCI, possibly because IPCI is a primary care database while many JAKi indications are specialist‐managed. Future JAKi safety studies may therefore benefit from specialist healthcare data. Recorded comorbidity completeness may also vary by database because of differences in healthcare setting and follow‐up. Differences in data capture, including primary‐care versus linked‐care coverage and prescription versus dispensing records, as well as differences in specialist‐care availability, underlying population characteristics and duration of follow‐up, may contribute to variation in the incidence estimates observed across data sources. Therefore, differences in estimates between data sources should not be interpreted as necessarily reflecting true differences in JAKi utilisation between countries. The findings would be more appropriately interpreted as temporal trends within each data source, with cross‐data‐source comparisons made cautiously and in the context of the underlying healthcare and data settings.

Despite these limitations, this study provides a comprehensive overview of JAKi use across European countries and care settings, informing the feasibility and design of future safety studies within DARWIN EU. The current study estimated the incidence of JAKi use in the general population rather than within populations defined by specific indications. Evaluating indication‐specific incidence, for example among individuals with rheumatoid arthritis, inflammatory bowel disease or atopic dermatitis, could provide additional insights into differences in JAKi uptake and utilisation patterns across indicated populations and represents an important direction for future research. The current study was also not designed to formally evaluate the impact of emerging safety evidence or subsequent regulatory actions on JAKi utilisation. Given that the ORAL Surveillance findings and subsequent EMA safety recommendations occurred towards the end of the study period, which extended only through 2023, there was limited post‐regulatory follow‐up available to assess potential changes in utilisation. In addition, the descriptive design of the current study did not allow observed changes in JAKi uptake to be attributed to specific regulatory actions. Future studies with longer follow‐up could formally evaluate the impact of regulatory safety communications and risk‐minimisation measures on JAKi prescribing and utilisation, for example, using interrupted time‐series approaches. Given that the EMA recommendations specifically identified older individuals, including those aged ≥ 65 years, as a population requiring additional consideration when prescribing JAKi, future studies could also examine temporal trends stratified by age (e.g., ≥ 65 vs. < 65 years) before and after the emergence of the ORAL Surveillance findings and subsequent regulatory recommendations. Such analyses could provide further insight into whether JAKi utilisation patterns changed differently among younger and older individuals following the emergence of safety evidence and implementation of regulatory risk‐minimisation measures. Future studies could additionally evaluate JAKi utilisation patterns among individuals with specific cardiovascular or thromboembolic risk factors. This would be particularly relevant given the safety concerns regarding MACE identified in the ORAL Surveillance study and subsequent regulatory review.

## 5 Conclusions

JAKi incidence increased between 2017 and 2023, with a temporary decline during the COVID‐19 pandemic. Tofacitinib was the most initiated JAKi. Except for abrocitinib, initiators were predominantly middle‐aged and female, with rheumatoid arthritis the most common indicated condition; atopic dermatitis predominated for abrocitinib. Median treatment duration was generally less than 1 year and some within‐class switching was observed. This study describes indication distribution and JAKi treatment patterns, providing a framework for future research.

## Author Contributions

X.C., D.P.‐A., A.B., A.J., A.P., A.S.‐M.L. and E.B. contributed to study design. X.C., A.S.‐M.L. and E.B. performed the data analysis. X.C. and A.S.‐M.L. drafted the first manuscript. G.C. contributed to phenotype review. T.L. contributed as data partners for FinOMOP‐HILMO; K.V. for IPCI; D.V. for IQVIA DA Germany; S.H., H.N. and N.T.H.T. for NLHR and G.S.‐G. for VID. Data partners did not have an investigator role; they executed analytical code at their respective data sources, reviewed and approved their results. All authors contributed to data interpretation, critically revised the manuscript for important intellectual content and approved the final version.

## Funding

This study was funded by the European Medicines Agency (EMA) and performed via DARWIN EU (EUPAS1000000424). EMA was involved in conceiving the research objectives and reviewing the study protocol and study report, including the results. This communication represents the DARWIN EU Coordination Centre only and cannot be interpreted as reflecting the views of the EMA or the European Medicines Regulatory Network. The views expressed in this article are the personal views of the authors and may not be understood or quoted as being made on behalf of or reflecting the position of, the regulatory agency/agencies or organisations with which the authors are employed or affiliated.

## Ethics Statement

Ethics approval was obtained in each data source from the respective ethics committee: the Clinical Research Ethics committee of Terveyden ja hyvinvoinnin laitos (THL) for FinOMOP‐HILMO (application number 9.12.2024); the IPCI Review Board for IPCI (application number 10/2022 and 11/2022), Regionale komiteer for medisinsk og helsefaglig forskningsetikk (REK) for NLHR (application number 155294); Comité Ético de Investigación con Medicamentos del Hospital Clínico Universitario de Valencia for VID (application number 2024/421). No ethics approval was required for IQVIA DA Germany as the database was anonymised in nature.

## Consent

The authors have nothing to report.

## Conflicts of Interest

Prof. Daniel Prieto‐Alhambra is Principal Investigator for Real World Evidence studies sponsored by UCB Biopharma and Theramex and a co‐investigator for a study sponsored by Eli Lilly and previous co‐investigator for a study sponsored by Novartis. He has also been a co‐investigator in IMI‐funded projects (EHDEN, EU‐PEARL) and is currently involved in OPTIMA ONCOLOGY, while leading the IHI‐funded GREG (ihi‐greg.eu) programme. His research group has received public funding from NIHR and the European Medicines Agency. Prof. Prieto‐Alhambra is a member of the ENCePP Steering Group. The other authors declare no conflicts of interest.

## Supporting information


**Table S1:** Year of marketing authorisation approval and indications of JAKi by EMA.
**Table S2:** Overview of data sources in the study.
**Table S3:** Codelist for identifying JAKi and indicated conditions.
**Table S4:** Attrition table for identifying denominator population.
**Table S5:** Number of individual JAKi ingredient initiators identified for incidence analysis.
**Figure S1:** Incidence of JAKi use among the individuals without prior exposure to the any JAKi.

## Data Availability

Individual‐level data are not available for this study. Analytical codes are available at https://github.com/darwin‐eu‐studies/EUPAS1000000424. The full set of results for this study is available through an interactive web‐application Shiny app at https://data.darwin‐eu.org/EUPAS1000000424/.
