# Supplementary material for: Temporal Trends in Janus Kinase Inhibitor (JAKi) Use Across Five European Countries: A DARWIN EU Network Study
Source: Pharmacoepidemiol Drug Saf. 2026 Oct 14;35(11):e70502. doi: 10.1002/pds.70502 (PMC13664646; doi:10.1002/pds.70502)
Supplement: Supplementary file 1 [file PDS-35-e70502-s001.docx]

**Temporal trends in Janus kinase inhibitor (JAKi) use across five European countries: a DARWIN-EU network study**

Xihang Chen, Andrei Barbulescu, George Corby, Saeed Hayati, Annika Joedicke, Toni Lehtonen, Hedvig Marie Egeland Nordeng, Alexandra Pacurariu, Gabriel Sanfélix-Gimeno, Nhung TH Trinh, Katia Verhamme, Dina Vojinovic, Daniel Prieto-Alhambra, Amy Shuk-Man Lam*, Edward Burn*

**Supplementary tables and figures**

Table of Contents

[Supplementary Table S1: Year of marketing authorisation approval and indications of JAKi by EMA	2](#_Toc228899296)

[Supplementary Table S2: Overview of data sources in the study	2](#_Toc228899297)

[Supplementary Table S3. Codelist for identifying JAKi and indicated conditions.	4](#_Toc228899298)

[Supplementary Table S4. Attrition table for identifying denominator population.	7](#_Toc228899299)

[Supplementary Table S5. Number of individual JAKi ingredient initiators identified for incidence analysis.	10](#_Toc228899300)

[Supplementary Figure S1: Incidence of JAKi use among the individuals without prior exposure to the any JAKi.	12](#_Toc228899301)

# Supplementary Table S1: Year of marketing authorisation approval and indications of JAKi by EMA

| **Ingredient** | **Baricitinib** | **Tofacitinib** | **Upadacitinib** | **Filgotinib** | **Abrocitinib** |
| --- | --- | --- | --- | --- | --- |
| **Year of marketing authorisation approval** | 2017 | 2017 | 2019 | 2020 | 2021 |
| **Approved indication** | - Alopecia areata - Atopic dermatitis - Juvenile idiopathic arthritis - RA | - Axial spondylitis - Juvenile idiopathic arthritis - PsA - RA - UC | - Atopic dermatitis - Axial spondylitis - IBD - PsA - RA | - RA - UC | - Atopic dermatitis |

Abbreviation: EMA=European Medicines Agency; IBD=inflammatory bowel disease; JAKi=Janus kinase inhibitor; PsA=psoriatic arthritis; RA=rheumatoid arthritis; UC=ulcerative colitis.

# Supplementary Table S2: Overview of data sources in the study

| Database | Finland (FinOMOP-HILMO) | Germany (IQVIA DA Germany) | Norway (NLHR) | Spain (VID) | Netherlands (IPCI) |
| --- | --- | --- | --- | --- | --- |
| Type of data source | Public and private primary and specialised inpatient/outpatient healthcare registries + prescriptions + vaccinations | Ambulatory EHR from GPs and specialists | Linked national health registries | Integrated regional healthcare database / EHR | Primary care GP EHR |
| Primary care | Diagnosis, prescription | Diagnosis, Prescribed prescription, Procedures | Diagnosis, Dispensed prescription | Diagnosis, Prescribed & prescription | Diagnosis, Lab, Lifestyle, prescription |
| Specialist outpatient care | Diagnosis, Procedures, prescription | Diagnosis, Prescribed prescription, Procedures | Diagnosis, Dispensed prescription | Diagnosis, Procedures, prescription, Lab | Referral/discharge letters only |
| Inpatient specialist care / hospital care | Diagnosis, Procedures, Vaccinations | Not covered | Diagnosis | Diagnosis, Procedures, prescription, Lab | Hospital information via correspondence only |
| Data availability in analyses | ~2011–2023 (full coverage) | 1989-2023 | 2008-2023 (primary care data), 2018-2023 (secondary care data and drug dispensation data) | 2018-2021 | 2006-2024 |
| Individuals in data source / eligible | Nationwide Finnish population | Nationwide database covering 45M | Nationwide database covering ~5.5M | Valencia regional population | ~350 GP practices (~7% Dutch population) |
| Limitations relevant to study | No dispensing/use confirmation; incomplete private care before 2020; no reliable dose/days supply | Follow-up defined earliest-to-latest event; no linkage across GPs | Missing some diagnostic codes; no procedures; no dose/route; only selected expensive drugs given in hospital are included; no OTC records | Regional coverage only; specialist conditions may be incompletely captured. The database only contains females aged from 8 to 59 | Limited hospital detail; no coded hospital medications/procedures/tests |
| Impact of limitations | Possible exposure misclassification and time-trend artefacts | Artificial inflation of incidence/prevalence near data cut; mitigated by stopping follow-up 6 months before cut | Broad population coverage but underestimation of comorbidities and inpatient drug treatment | May underestimate diseases mainly managed outside regional/public system. The database is underrepresented in certain demographics | Under-capture of specialist/hospital treatments and outcomes |

**Abbreviations:** EHR: Electronic health care records, IPCI: Integrated Primary Care Information NLHR: Norwegian Linked healthcare registries.

# Supplementary Table S3. Codelist for identifying JAKi and indicated conditions.

| Name | Concept id |
| --- | --- |
| JAKi | |
| JAKi | 36922597, 1758974, 1758986, 36952118, 1758991, 1758989, 1758982, 36930705, 36941240, 1758993, 1758988, 1758990, 1758976, 36927189, 1758984, 1758985, 36920251, 1758987, 1758992, 1758975, 1758977, 36935695, 36946300, 36963824, 1758978, 1758981, 36949742, 36947156, 1758979, 36947759, 1758983, 36506007, 36787583, 40743760, 40743761, 36508742, 36787575, 36504859, 42875510, 2920717, 36787585, 36787579, 40743762, 1510630, 36787587, 779707, 1510638, 44185375, 36787582, 1510628, 42875511, 36508730, 36787572, 44187244, 36787574, 1510631, 1758613, 36787586, 1758612, 37497354, 36787577, 994801, 36504599, 42875513, 37497355, 36787591, 36787588, 1510637, 37497353, 42875514, 44187243, 1510627, 36503766, 36787589, 36787584, 44166708, 36421026, 1510632, 36787578, 36787581, 36509872, 779706, 42875515, 2920716, 1510629, 44169060, 37497352, 37592678, 1510635, 994802, 36787537, 36787576, 36787580, 36787573, 36508651, 1510636, 2056664, 1510634, 36787590, 44180263, 42875512, 36036733, 35896991, 35887301, 35896989, 35896993, 35896990, 35887307, 36036735, 35896998, 36036734, 35887303, 35891916, 35887308, 35887309, 35887300, 35896996, 35896994, 35896997, 35896995, 35887305, 35887302, 36036738, 35896992, 36036737, 35887310, 35896999, 35887306, 36036736, 35887304, 36953781, 36934485, 36946261, 36935408, 36788672, 43025851, 1830306, 1830308, 36788670, 35605688, 42904205, 36788677, 36788665, 36960395, 1559832, 37498556, 35605683, 2930688, 37498555, 1830302, 36421481, 36508461, 36067671, 1559833, 36951570, 36788681, 1970541, 2930690, 36788676, 43280270, 42901964, 36507751, 739797, 36509967, 36788679, 36933989, 36943391, 739798, 36788673, 42875334, 739800, 36788666, 36035120, 36035119, 1830301, 37592375, 36508669, 43025850, 42901327, 1830309, 36788664, 1830305, 36067669, 36504178, 36246799, 36246801, 1830303, 42656016, 37498558, 1970540, 36788674, 1559835, 36246800, 42901965, 35605686, 36814486, 739801, 42656011, 36067668, 1970546, 36246802, 42901324, 1970543, 36788669, 37498557, 36421480, 36788680, 2930692, 36812958, 35605685, 739796, 36067670, 1970545, 2930689, 2930687, 42875333, 42656012, 21105873, 37592376, 42656015, 36788678, 35605684, 36788667, 36509806, 739802, 36035121, 36812392, 44074810, 42656014, 42901323, 1830307, 739799, 36969044, 1970539, 36035122, 37592374, 1970542, 2930691, 35605687, 43025852, 739795, 36504798, 1970544, 36788671, 42901326, 42656013, 43025849, 1559834, 35605682, 36035118, 1830310, 36961308, 37499794, 37499793, 1970547, 1830304, 44189253, 35605689, 43285737, 43025853, 42683528, 36788675, 2921732, 1830867, 1830868, 36074830, 1361602, 1971258, 1830865, 1361604, 36946517, 36065552, 1758760, 1361597, 1830862, 2921731, 1971257, 1758759, 1759248, 780232, 36923472, 1830863, 1830869, 779261, 1361580, 1361603, 1361707, 1361596, 36968864, 1830871, 1759249, 36035858, 1830864, 1830870, 36968418, 36035857, 36933601, 36961320, 779263, 1361600, 36065551, 36065553, 1361595, 1361598, 36035856, 36927195, 1361605, 36948574, 1830866, 1361601, 779262, 1361708, 779264, 1830872, 1758758, 1758761, 780231 |
| abrocitinib | 36922597, 1758974, 1758986, 36952118, 1758991, 1758989, 1758982, 36930705, 36941240, 1758993, 1758988, 1758990, 1758976, 36927189, 1758984, 1758985, 36920251, 1758987, 1758992, 1758975, 1758977, 36935695, 36946300, 36963824, 1758978, 1758981, 36949742, 36947156, 1758979, 36947759, 1758983 |
| baricitnib | 36506007, 36787583, 40743760, 40743761, 36508742, 36787575, 36504859, 42875510, 2920717, 36787585, 36787579, 40743762, 1510630, 36787587, 779707, 1510638, 44185375, 36787582, 1510628, 42875511, 36508730, 36787572, 44187244, 36787574, 1510631, 1758613, 36787586, 1758612, 37497354, 36787577, 994801, 36504599, 42875513, 37497355, 36787591, 36787588, 1510637, 37497353, 42875514, 44187243, 1510627, 36503766, 36787589, 36787584, 44166708, 36421026, 1510632, 36787578, 36787581, 36509872, 779706, 42875515, 2920716, 1510629, 44169060, 37497352, 37592678, 1510635, 994802, 36787537, 36787576, 36787580, 36787573, 36508651, 1510636, 2056664, 1510634, 36787590, 44180263, 42875512 |
| filgotinib | 36036733, 35896991, 35887301, 35896989, 35896993, 35896990, 35887307, 36036735, 35896998, 36036734, 35887303, 35891916, 35887308, 35887309, 35887300, 35896996, 35896994, 35896997, 35896995, 35887305, 35887302, 36036738, 35896992, 36036737, 35887310, 35896999, 35887306, 36036736, 35887304, 36953781, 36934485, 36946261, 36935408 |
| tofacitinib | 36788672, 43025851, 1830306, 1830308, 36788670, 35605688, 42904205, 36788677, 36788665, 36960395, 1559832, 37498556, 35605683, 2930688, 37498555, 1830302, 36421481, 36508461, 36067671, 1559833, 36951570, 36788681, 1970541, 2930690, 36788676, 43280270, 42901964, 36507751, 739797, 36509967, 36788679, 36933989, 36943391, 739798, 36788673, 42875334, 739800, 36788666, 36035120, 36035119, 1830301, 37592375, 36508669, 43025850, 42901327, 1830309, 36788664, 1830305, 36067669, 36504178, 36246799, 36246801, 1830303, 42656016, 37498558, 1970540, 36788674, 1559835, 36246800, 42901965, 35605686, 36814486, 739801, 42656011, 36067668, 1970546, 36246802, 42901324, 1970543, 36788669, 37498557, 36421480, 36788680, 2930692, 36812958, 35605685, 739796, 36067670, 1970545, 2930689, 2930687, 42875333, 42656012, 21105873, 37592376, 42656015, 36788678, 35605684, 36788667, 36509806, 739802, 36035121, 36812392, 44074810, 42656014, 42901323, 1830307, 739799, 36969044, 1970539, 36035122, 37592374, 1970542, 2930691, 35605687, 43025852, 739795, 36504798, 1970544, 36788671, 42901326, 42656013, 43025849, 1559834, 35605682, 36035118, 1830310, 36961308, 37499794, 37499793, 1970547, 1830304, 44189253, 35605689, 43285737, 43025853, 42683528, 36788675 |
| upadacitinib | 2921732, 1830867, 1830868, 36074830, 1361602, 1971258, 1830865, 1361604, 36946517, 36065552, 1758760, 1361597, 1830862, 2921731, 1971257, 1758759, 1759248, 780232, 36923472, 1830863, 1830869, 779261, 1361580, 1361603, 1361707, 1361596, 36968864, 1830871, 1759249, 36035858, 1830864, 1830870, 36968418, 36035857, 36933601, 36961320, 779263, 1361600, 36065551, 36065553, 1361595, 1361598, 36035856, 36927195, 1361605, 36948574, 1830866, 1361601, 779262, 1361708, 779264, 1830872, 1758758, 1758761, 780231 |
| Indicated conditions | |
| alopecia_areata | 141933, 4056343, 4312756, 4297823, 42596259, 4291286, 4300913, 4300912, 4065243, 4300910, 4031164, 4300094, 4263194, 4299701, 4300911, 42598468, 4300914, 4239312 |
| atopic_dermatitis | 4236759, 4031630, 4031013, 4296192, 4033671, 4080929, 4223641, 4290736, 4297478, 4297494, 4002519, 4296193, 4223478, 4298601, 4297362, 4297495, 4298600, 133834, 4321570, 4290737, 40482226, 4290738, 4206125, 4031012, 4031634, 4290740, 4031016, 4298598, 4296190, 4033672, 4033770, 4066727, 4080927, 1340257, 4290734, 4080928, 4298597, 4298599, 4296191, 4066382, 4210912 |
| axial_spondylitis | 4035614, 36716891, 607398, 3654715, 1340249, 4083681, 4079735, 37017494, 437082, 37203959, 4035741 |
| inflammatory_bowel_disease | 602593, 4031048, 40479839, 194077, 40482241, 1340491, 46269951, 40479837, 46269838, 4025853, 4301738, 81893, 78799, 40482865, 4302002, 4187900, 4166480, 602594, 602607, 46269848, 4086978, 44783784, 77317, 4029372, 4342656, 40481367, 4259504, 1340490, 36715915, 201606, 36716986, 4142544, 4055020, 36686095, 4055884, 4323289, 4239382, 46269878, 4264850, 46274073, 46269888, 36716695, 4212992, 4266370, 194684, 42537666, 1340297, 4242392, 4210469, 46269883, 4212991, 4244235, 4342643, 37116446, 4246693, 195575, 195585, 4177488, 4122617, 4340114, 46269880, 46269881, 46269882, 46269879, 4131542, 46269891, 46269876, 46269875, 46269874, 46269886, 46269877, 46269885, 46269884, 46269887, 46269890, 46269889, 4074815, 4116142, 4116143, 75580 |
| juvenile_arthritis | 608034, 608035, 1340385, 42535177, 42535150, 608043, 608044, 608042, 75621, 608041, 608076, 4116447, 4211939, 608094, 608095, 608092, 37204149, 608093, 4261026, 4132810, 4257971, 4132809, 603150, 72714, 75622, 72705, 4035612, 4079736, 4079731, 4259507, 4083558, 608075, 762178, 4115377, 762180, 4253902, 4114447, 4261027, 4132811, 37207517, 4132812, 4257972, 4079733, 4079734, 4083680, 4253901, 37209479, 37204148 |
| juvenile_idiopathic_arthritis | 4132810, 4257971, 4079731 |
| psoriatic_arthritis | 4025831, 46274123, 4079734, 4035742, 81931, 4132495, 4083682, 1340448, 1340520, 4064048, 40319772 |
| rheumatoid_arthritis | 46273442, 44811155, 44811154, 44811153, 44811152, 44811151, 44811073, 44808003, 44790163, 42689682, 42689681, 42689680, 42689679, 42539550, 42536657, 42534841, 42534840, 42534839, 42534838, 42534837, 42534836, 42534835, 42534834, 40484633, 40482696, 37395590, 37309724, 37309723, 37309722, 37209329, 37209328, 37209323, 37209322, 37209321, 37207810, 37207809, 37207808, 37207807, 37207806, 37207805, 37207804, 37108714, 37108591, 37108590, 36687006, 36687005, 36687003, 36687002, 36687001, 36687000, 36686999, 36685024, 36685023, 36685022, 36685021, 36685020, 36685019, 36685018, 36685017, 36684998, 36684997, 36683391, 36304514, 35609010, 35609009, 4334806, 4330635, 4311391, 4297650, 4296152, 4271003, 4243205, 4216531, 4179536, 4179528, 4179378, 4147418, 4117687, 4117686, 4116446, 4116445, 4116444, 4116443, 4116442, 4116441, 4116440, 4116439, 4116153, 4116152, 4116151, 4116150, 4116149, 4116148, 4115161, 4115051, 4115050, 4114444, 4114442, 4114441, 4114440, 4114439, 4107913, 4102493, 4083556, 4078299, 4060405, 4035611, 4035427, 2108722, 2108721, 2108720, 2108719, 2107572, 2107561, 2107560, 2107559, 2107558, 1176038, 256197, 81097, 80809, 40481048, 40481959, 40482839, 4028118, 3654567 |

# Supplementary Table S4. Attrition table for identifying denominator population.

| Reason | **Variable name** | | | |
| --- | --- | --- | --- | --- |
|  | Number records | Number subjects | Excluded records | Excluded subjects |
| **FinOMOP-HILMO** | | | | |
| Starting population | 6,696,420 | 6,618,745 | - | - |
| Missing year of birth | 6,696,420 | 6,618,745 | 0 | 0 |
| Missing sex | 6,695,862 | 6,618,208 | 558 | 537 |
| Cannot satisfy age criteria during the study period based on year of birth | 6,695,862 | 6,618,208 | 0 | 0 |
| No observation time available during study period | 6,285,816 | 6,251,046 | 410,046 | 367,162 |
| Doesn't satisfy age criteria during the study period | 6,285,816 | 6,251,046 | 0 | 0 |
| Prior history requirement not fulfilled during study period | 6,157,296 | 6,133,938 | 128,520 | 117,108 |
| No observation time available after applying age, prior observation and, if applicable, target criteria | 6,157,296 | 6,133,938 | 0 | 0 |
| Starting analysis population | 6,157,296 | 6,133,938 | - | - |
| Apply washout and censor cohort - anyone with outcome prior to start excluded | 6,157,285 | 6,133,929 | 11 | 9 |
| **IPCI** | | | | |
| Starting population | 2,954,616 | 2,954,616 | - | - |
| Missing year of birth | 2,954,616 | 2,954,616 | 0 | 0 |
| Missing sex | 2,954,616 | 2,954,616 | 0 | 0 |
| Cannot satisfy age criteria during the study period based on year of birth | 2,954,616 | 2,954,616 | 0 | 0 |
| No observation time available during study period | 2,254,608 | 2,254,608 | 700,008 | 700,008 |
| Doesn't satisfy age criteria during the study period | 2,254,608 | 2,254,608 | 0 | 0 |
| Prior history requirement not fulfilled during study period | 2,016,848 | 2,016,848 | 237,760 | 237,760 |
| No observation time available after applying age, prior observation and, if applicable, target criteria | 2,016,848 | 2,016,848 | 0 | 0 |
| Starting analysis population | 2,016,848 | 2,016,848 | - | - |
| Apply washout and censor cohort - anyone with outcome prior to start excluded | 2,016,752 | 2,016,752 | 96 | 96 |
| **IQVIA-DA Germany** | | | | |
| Starting population | 44,581,708 | 44,581,708 | - | - |
| Missing year of birth | 44,581,708 | 44,581,708 | 0 | 0 |
| Missing sex | 44,553,378 | 44,553,378 | 28,330 | 28,330 |
| Cannot satisfy age criteria during the study period based on year of birth | 44,535,086 | 44,535,086 | 18,292 | 18,292 |
| No observation time available during study period | 28,248,941 | 28,248,941 | 16,286,145 | 16,286,145 |
| Doesn't satisfy age criteria during the study period | 28,248,941 | 28,248,941 | 0 | 0 |
| Prior history requirement not fulfilled during study period | 14,312,751 | 14,312,751 | 13,936,190 | 13,936,190 |
| No observation time available after applying age, prior observation and, if applicable, target criteria | 14,312,751 | 14,312,751 | 0 | 0 |
| Starting analysis population | 14,312,751 | 14,312,751 | - | - |
| Apply washout and censor cohort - anyone with outcome prior to start excluded | 14,311,724 | 14,311,724 | 1,027 | 1,027 |
| **NLHR** | | | | |
| Starting population | 6,148,772 | 6,114,138 | - | - |
| Missing year of birth | 6,148,772 | 6,114,138 | 0 | 0 |
| Missing sex | 6,148,772 | 6,114,138 | 0 | 0 |
| Cannot satisfy age criteria during the study period based on year of birth | 6,148,772 | 6,114,138 | 0 | 0 |
| No observation time available during study period | 6,083,991 | 6,061,141 | 64,781 | 52,997 |
| Doesn't satisfy age criteria during the study period | 6,083,991 | 6,061,141 | 0 | 0 |
| Prior history requirement not fulfilled during study period | 5,852,116 | 5,834,891 | 231,875 | 226,250 |
| No observation time available after applying age, prior observation and, if applicable, target criteria | 5,852,116 | 5,834,891 | 0 | 0 |
| Starting analysis population | 5,852,116 | 5,834,891 | - | - |
| Apply washout and censor cohort - anyone with outcome prior to start excluded | 5,852,102 | 5,834,880 | 14 | 11 |
| **VID** | | | | |
| Starting population | 1,964,588 | 1,964,588 | - | - |
| Missing year of birth | 1,964,588 | 1,964,588 | 0 | 0 |
| Missing sex | 1,964,588 | 1,964,588 | 0 | 0 |
| Cannot satisfy age criteria during the study period based on year of birth | 1,964,588 | 1,964,588 | 0 | 0 |
| No observation time available during study period | 1,964,588 | 1,964,588 | 0 | 0 |
| Doesn't satisfy age criteria during the study period | 1,964,588 | 1,964,588 | 0 | 0 |
| Prior history requirement not fulfilled during study period | 1,573,684 | 1,573,684 | 390,904 | 390,904 |
| No observation time available after applying age, prior observation and, if applicable, target criteria | 1,573,684 | 1,573,684 | 0 | 0 |
| Starting analysis population | 1,573,684 | 1,573,684 | - | - |
| Apply washout and censor cohort - anyone with outcome prior to start excluded | 1,573,602 | 1,573,602 | 82 | 82 |

# Supplementary Table S5. Number of individual JAKi ingredient initiators identified for incidence analysis.

| Year^a^ | FinOMOP-HILMO | IPCI | IQVIA-DA Germany | NLHR | VID |
| --- | --- | --- | --- | --- | --- |
|  | abrocitinib |  |  |  |  |
| 2017 | 0 | 0 | 0 | - | - |
| 2018 | 0 | 0 | 0 | - | - |
| 2019 | 0 | 0 | 0 | 0 | 0 |
| 2020 | 0 | 0 | 0 | 0 | 0 |
| 2021 | 0 | 0 | 0 | 0 | 0 |
| 2022 | 0 | 6 | 0 | 219 | - |
| 2023 | 0 | 6 | - | 79 | - |
| Overall | 0 | 12 | 0 | 298 | 0 |
|  | baricitinib |  |  |  |  |
| 2017 | 85 | 11 | 39 | - | - |
| 2018 | 376 | 15 | 54 | - | - |
| 2019 | 261 | 12 | 110 | 518 | 7 |
| 2020 | 162 | 21 | 110 | 406 | 10 |
| 2021 | 124 | 16 | 109 | 271 | 13 |
| 2022 | 127 | 26 | 109 | 94 | - |
| 2023 | 63 | 7 | - | 193 | - |
| Overall | 1198 | 108 | 531 | 1482 | 30 |
|  | filgotinib |  |  |  |  |
| 2017 | 0 | 0 | 0 | - | - |
| 2018 | 0 | 0 | 0 | - | - |
| 2019 | 0 | 0 | 0 | 0 | 0 |
| 2020 | 0 | 0 | 15 | 0 | 0 |
| 2021 | 22 | 20 | 211 | 449 | <5 |
| 2022 | 53 | 24 | 366 | 602 | - |
| 2023 | 81 | 22 | - | 509 | - |
| Overall | 156 | 66 | 592 | 1560 | 0 |
|  | tofacitinib |  |  |  |  |
| 2017 | 87 | 11 | 141 | - | - |
| 2018 | 456 | 30 | 339 | - | - |
| 2019 | 489 | 43 | 464 | 136 | 64 |
| 2020 | 297 | 44 | 357 | 59 | 59 |
| 2021 | 338 | 32 | 152 | 24 | 37 |
| 2022 | 298 | 27 | 77 | 165 | - |
| 2023 | 160 | 18 | - | 258 | - |
| Overall | 2125 | 205 | 1530 | 642 | 160 |
|  | upadacitinib |  |  |  |  |
| 2017 | 0 | 0 | 0 | - | - |
| 2018 | 0 | 0 | 0 | - | - |
| 2019 | 0 | 0 | 0 | 0 | 0 |
| 2020 | 94 | <5 | 387 | <5 | <5 |
| 2021 | 286 | 10 | 661 | 114 | 113 |
| 2022 | 298 | 20 | 836 | 177 | - |
| 2023 | 618 | 32 | - | 252 | - |
| Overall | 1296 | 62 | 1884 | 543 | 113 |

Abbreviation: FinOMOP-HILMO=Finnish Care Register for Health Care; IPCI=Integrated Primary Care Information; IQVIA DA Germany=IQVIA Disease Analyzer Germany; JAKi=Janus kinase inhibitor; NLHR=Norwegian Linked Health Registry; VID=Valencia Health System Integrated Dataset.

a. Number of JAKi initiators identified for incidence analysis considered full-year contribution across all data sources, and drug record availability since 2018 in NLHR.

# Supplementary Figure S1: Incidence of JAKi use among the individuals without prior exposure to the any JAKi.

Abbreviation: FinOMOP-HILMO=Finnish Care Register for Health Care; IPCI=Integrated Primary Care Information; IQVIA DA Germany=IQVIA Disease Analyzer Germany; JAKi=Janus kinase inhibitor; NLHR=Norwegian Linked Health Registry; VID=Valencia Health System Integrated Dataset.
